# Comparative Assessment of the Fenestration, Dehiscence Frequency and Facial Bone Thickness in the Maxillary Anterior Region of Smokers Versus Non-Smokers: A Cross-Sectional Study

**DOI:** 10.1155/ijod/9783707

**Published:** 2025-04-19

**Authors:** Ferena sayar, Amirhossein Farahmand, Bahar Ebrahimi kordasiabi, Ladan Hafezi, Mahsa soleimani

**Affiliations:** ^1^Department of Periodontics, Faculty of Dentistry, Tehran Medical Sciences, Islamic Azad University, Tehran, Iran; ^2^Department of Periodontics, Faculty of Dentistry, Borujerd Medical Sciences, Islamic Azad University, Lorestan, Iran; ^3^Department of Oral Radiology, Faculty of Dentistry, Tehran Medical Sciences, Islamic Azad University, Tehran, Iran

**Keywords:** buccal bone thickness, dehiscence, fenestration, smoking

## Abstract

**Aim:** To investigate the presence of fenestration and dehiscence. In addition, the thickness of the facial bone in the anterior region of the maxillary anterior teeth was measured using C.B.C.T. scans in both smokers and non-smokers.

**Materials and Methods:** The study used 300 C.B.C.T. Scans of patients with intact maxillary anterior teeth to assess the presence of bone defects in the fenestration and dehiscence areas. In addition, the thickness of the facial bone in the anterior region of the maxilla was measured in both smokers and non-smokers.

**Results:** The study included 300 participants free of systemic or periodontal diseases. The subjects were divided into smokers (49.33%) and non-smokers (50.66%). The results showed that female smokers most frequently had fenestrations in the right premolar and left canine areas, with a majority rate of 1.57%. However, dehiscence was most common in male smokers, with the left premolar area being the most common with a frequency of 3.43%.

**Conclusion:** The study found that smoking reduced the thickness of the labial bone in the anterior maxillary regions. In addition, female smokers had a higher frequency of fenestrations in the right premolar and left canine regions, and on average, smoking patients had the highest rate of dehiscence in the left premolar–maxillary region.

## 1. Introduction

The two most common types of alveolar defects are dehiscence and fenestration. Alveolar dehiscence is the absence of facial or lingual alveolar cortical plates. A circumscribed defect in the cortical plate exposes the underlying root surface but does not involve the alveolar of the bone [1–3]. These defects may extend the entire length of the tooth surface root or be as small as one or two millimetres. Dehiscence leads to gingival recession, alveolar bone resorption and tooth root exposure. At the same time, fenestration is a 'window' of alveolar bone defects in the buccal or lingual surface of the tooth, exposing the root surface directly to contact with the gingival or alveolar mucosa [4]. Although dehiscence and fenestration are non-pathological situations, they result in variations in the periodontal tissue area. The common, undiagnosed or unexpected presence of these conditions can complicate periodontal surgical procedures, lead to changes in implant surgical protocols and complicate treatment [2]. Careful monitoring and control of alveolar bone dehiscence and fenestration can prevent adverse effects on periodontal treatment. To develop an effective treatment plan, it is important to understand the differences between these two defects and their impact on local root exposure. Further research and advances in periodontal therapy will allow us to improve outcomes and better care for patients with these conditions [5, 6]. According to a study by Evangelista et al. [7], 51.09% of the alveolar bone defects were dehiscence and 36.51% were fenestrations. Similarly, Sun et al. [3] reported that 61.57% of alveolar bone defects were dehiscence and 31.93% were fenestrations in the mandibular arch. These results highlight the prevalence of these defects and the importance of their detection and treatment in periodontal and implant surgery. The purpose of this study was to determine the frequency of alveolar bone dehiscence and fenestration and to measure the degree of alveolar bone loss and thickness or overlay of the maxillary anterior teeth in individuals with Class I malocclusion. This was done using cone beam computed tomography (C.B.C.T.).

## 2. Materials and Methods

### 2.1. Study Population

The study was conducted at the Department of Periodontology, Medical Science Dental School of Dentistry, Islamic Azad University, Tehran, during 2021–22. The study population consisted of two groups: smokers and non-smokers. C.B.C.T. scans from patients who met the inclusion criteria were evaluated. The study was approved by the Ethics Committee of the Islamic Azad University of Medical Sciences, Dental School, Tehran/Iran (IR.IAU.Dental.REC.1399.172). The C.B.C.T. images of patients with intact maxillary facial bone structures of the anterior teeth were evaluated after reviewing all available images. Univariate and multivariate analyses were performed to examine the associations between fenestration and dehiscence as well as buccal bone thickness and age, gender and smoking habits.

### 2.2. Inclusion Criteria

Healthy patients—without periodontal disease in anterior maxillary teeth—over 25 years old—Class I molar relationship—intact right and left maxillary anterior teeth—more than 10 cigarettes per day for at least 1 year (amount of smoke as pack/year (number of cigarettes per day × years) of smoking) [8] will be calculated—willingness to participate in the study.

### 2.3. Exclusion Criteria

Systemic disease; being pregnant or breastfeeding; extensive tooth decay; record and present orthodontic treatment processes; periapical surgery record of periodontal surgery; use of removable prostheses; tooth abscess; taking any medication.

### 2.4. Sample Size

A minimum required sample size of 150 for each group was established based on previous studies and existing limitations, as well as observation by Nowzari et al. [9]. Using the option comparison of the two averages to determine the sample size with the Mini Tab software and taking into account *α* = 0.005, a power of 90%, as well as STD = 1.3 and *d* = 0.5, 300 subjects were evaluated in the current study.

### 2.5. Methods

**Figure 1:**
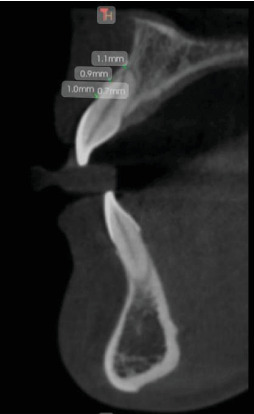
Facial thickness bone in anterior of maxillary regions.

**Figure 2:**
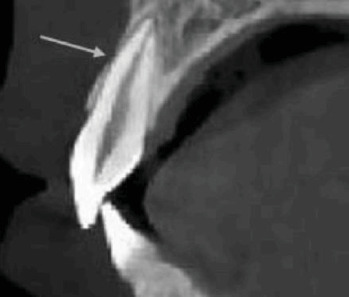
Fenestration in maxillary central incisor.

**Figure 3:**
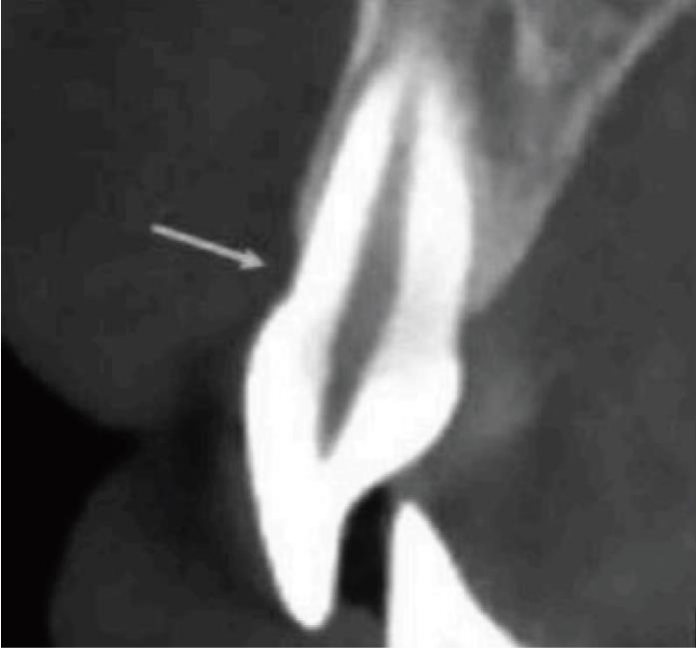
Dehiscence in maxillary central incisor.

The study employed a cross-sectional approach, with participants selected from patients referred to the periodontics department at Azad University of Medical Sciences in Tehran. Specifically, the focus was on those patients who were scheduled for implant treatment. From this pool of potential participants, 300 individuals were randomly chosen for the study. This group included both smokers and non-smokers, with at least 10 cigarettes being the minimum smoking frequency among the smoking subjects, moreover included participants who were chosen based on their non-smoking status and completed a questionnaire to provide personal and general information. Prior to the study, the subjects were asked to sign a written consent form. C.B.C.T. images were then taken using a Radiography Rotograph EVO 3D device with a voxel size of 0.2 mm and a standard field of view (FOV) measuring 8.5 × 8.5 cm will be used for maxilla imaging at the Dental School of the Department of Oral and Maxillofacial Radiology in Islamic Azad University of Medical Sciences. Each slice will have a thickness of 1 mm. The scans will include checks for fenestration and dehiscence in each patient. The buccal bone thickness will be measured starting from 4 mm of crestal alveolar bone (Figure 1). Additionally, cross-sectional and axial images will be taken to assess the presence or absence of fenestration and dehiscence. Following the reconstruction of volumetric data, cross-sectional images with a thickness of 1 mm and measurements in the bucco–palatal dimensions were generated. Two periodontologists and one maxillofacial radiologist independently assessed the cross-sectional and axial images to identify the presence or absence of fenestration and dehiscence (Figures 2 and 3). Furthermore, the locations and extent of the bony lesions were categorised according to the cervical, middle and apical thirds of the buccal surfaces. Fenestration was defined as a localised bone defect or exposure of the underlying alveolar bone on the root surface while maintaining intact marginal bone. Conversely, if the bone defect extended to the marginal bone, it was classified as dehiscence. In cases of uncertainty, the findings were determined based on a consensus agreement between the two observers. The three observers were maxillofacial radiologists with over a decade of experience in interpreting C.B.C.T. images. After 2 weeks, the observers re-evaluated all images to assess intra-observer reliability.

### 2.6. Statistical Analysis

One-way statistical variance tests (ANOVA) were used to statistically compare the alveolar bone defects of subjects in the smoking and non-smoking groups. The Tukey test was used to further analyse and compare the mean of these data in the two groups. To examine the effect of variables, a multiple linear regression test was used.

## 3. Results

### 3.1. Demographic Information

The study conducted a cross-sectional analysis of healthy 300 subjects (without periodontal disease) to determine the prevalence of fenestration and dehiscence. Demographic information showed that 59.3% (178 cases) of cases were from women and 40.7% (122 cases) were from men. The majority of patients (83.3%) were between 30 and 48 years old. One hundred forty-eight patients who were free of systemic and periodontal diseases took part in the survey, of which 49.33% were smokers. In addition, a total of 152 individuals, representing 50.66% of the samples, were identified as non-smokers. These individuals had an average age of 39.64 years.

### 3.2. Bone Thickness of the Maxillary Anterior Teeth

It was found that the bone thickness of the maxillary anterior teeth differs significantly between smokers and non-smokers. Specifically, the central incisors on both the right and left sides had a minimum thickness of 0.69 ± 0.01 and 0.70 ± 0.01, respectively, as well as thin buccal bone. The lateral incisors on the left side and the premolars on both sides of the maxillary segment also had a mean facial bone plate thickness of less than 2 mm (0.01–0.68). Interestingly, the men's upper right premolars had the smallest thickness at 0.01–0.60. Notably, there was significantly less average change in labial bone thickness in smokers compared to non-smokers, as shown in Table 1.

### 3.3. The Frequency of Fenestrations and Dehiscence in the Anterior Maxillary Teeth

Based on the data presented in Table 2, the area with the highest prevalence of fenestrations in smokers was found to be the right premolar and left canine, with a prevalence rate of 57.1%. In contrast, the left premolar region was most common among non-smokers (28.2%). Regarding fenestration frequency, male smokers and non-smokers had lower rates than female smokers in similar areas. For example, fenestration in the premolar area was absent in men, whereas the prevalence in similar cases in women was 22.5%. In addition, the results showed that smoking patients had, on average, the highest rate of dehiscence in the left premolar region (Table 3).

### 3.4. The Frequency of Fenestrations in the Front Teeth of the Maxilla According to Gender and Smoking Habits

According to the results of the logistic regression test and the data presented in Tables 4 and 5, it was observed that fenestrations occurred more frequently in women compared to men, with proportions of 7% and 2%, respectively. When examining the effects of smoking, it was found that the likelihood of fenestration was eight times higher in the right premolar area and four times higher in the left canine area (*p* < 0.1). In addition, it was found that smoking was twice as likely to cause fenestrations in the right premolar region. In addition, women were found to have a 2% higher incidence of fenestrations in the maxillary left premolar region than men. Other results showed a significant correlation between age and the prevalence of fenestration in the right central incisor. The variables examined in this study were the right central incisor and left lateral incisor, which did not influence the occurrence of fenestrations in the teeth and bilateral fenestrations in the maxilla and canines.

### 3.5. To Examine the Effect of Sex and Cigarette Consumption on the Occurrence of Dehiscence

The results of the logistic regression analysis are presented in Tables 6 and 7. The results suggest that smokers have a significantly higher of developing premolar dehiscence compared to non-smokers, with the risk being more than three times higher. In addition, the study found a strong association between the presence of alveolar bone dehiscence in the maxillary anterior teeth and the age of the patient and the right and left maxillary canines. However, it is important to note that none of the factors examined in this study had an influence on the prevalence of dehiscence in the right central incisor, right incisor and left incisor.

### 3.6. To Examine the Effects of Smoking, Gender and Smoking on Buccal Bone Thickness

The data obtained from linear regression tests were analysed to examine the effects of gender and smoking on facial bone thickness. The results presented in Tables 8 and 9 showed that gender had no significant influence on the thickness of the facial bones in the left and right premolar areas. However, in patients who smoked, there was a reduction in the thickness of the right premolar by 0.06 mm and the left premolar by 0.08 mm (*p* < 0.1). Furthermore, thickness reduction was observed in the central and lateral right incisors by 0.45 mm and in the canines and left incisors by 0.06 mm and 0.07 mm, respectively. In all of these regions, facial bone thickness was found to be greater in men than in women. Of note, the bone thickness of the right and left central canines was not affected by any of the variables analysed, and the same was also observed for the bone thickness of the anterior maxilla.

## 4. Discussion

Despite the advantages of C.B.C.T., there is a lack of research on the association between smoking and superimposed maxillary anterior alveolar bone defects. It is important to evaluate this association because smoking has been linked to a variety of oral health problems, including periodontitis and bone loss. By studying the effects of smoking on these specific bone defects, the dental practitioners can better understand the potential risks and develop more effective treatment plans. Ultimately, the recurrence of periodontal treatment or gingivitis may be attributed to the misdiagnosis of buccal alveolar bone plates. To solve this problem, fenestration of the maxillary arch is often used. This procedure is particularly common on the first molars and canines as well as in the front area of the lower jaw. It is worth mentioning that misdiagnosis occurs more frequently in the upper jaw, especially in the area of the first premolar. The occurrence in the canine area is also consistently observed in all groups [10]. In addition, Jing et al. [11] reported that maxillary facially positioned teeth in the bone casing, lateral incisors and canines were more likely to have alveolar fenestration. Therefore, these specific teeth were analysed in this research. Furthermore, Sun et al. [3] showed that 27.46% of the anterior teeth examined had dehiscence, while fenestration was observed in 26.91% of them. Severe openings and fenestrations were mostly observed in the lower canines and upper canines, respectively. Each patient had alveolar bone defects, with one patient having defects in 91.67% of the anterior teeth [3], as seen in the current study examining the left and right sides of the maxilla. Alkezman et al. [12] found that 80% of maxillary anterior teeth had root fenestration in the facial bone. Women had a higher proportion (57.5%) than men. The right lateral incisor was the tooth most frequently affected by fenestration at 35.0%. Type IV root fenestrations (fenestration on the apical and middle parts of the root) were most commonly observed, accounting for 52.5% of cases. No significant difference was observed between different windows and age categories (*p* < 0.365) and also like the present study, the mean window height of the left canine was recorded as 7.1 mm, whereas the right lateral incisor had the lowest measurement at 4.9 mm. Additionally, it is worth noting that the prevalence of lingual (palatal) fenestration reported in the literature was relatively low. In this study, tooth number 12 (right lateral incisor) was found to be the most frequently affected by fenestration, with a prevalence of 35.0%. Following closely was tooth number 13 (right canine) with a prevalence of 28.7% [12]. The fenestration in the first premolars may be due to the anatomical features visible on the facial plates. However, some believe that narrow zones of attached gingiva, high frenal attachments and teeth located toward the buccal surface are often associated with these defects. The results of this study suggest a higher incidence of fenestrations in premolar teeth, which is consistent with other analyses. Most of the fenestration was observed in the maxilla [4]. And the first premolars in the upper jaw are located in areas that become narrower towards the top [13]. Of note is the relationship between the prevalence of dehiscence and the thickness of the alveolar bone. Rupprecht et al. [14] and Nimigean et al. [6] conducted studies on bone defects in the maxilla and mandible, including the maxillary and mandibular first molars and the mandibular canines and posterior teeth. In a study by Tal [15], it was observed that the highest frequency of fenestration defects occurred in bilateral canines, which is inconsistent with the analysis. Pan et al. [8] also reported a higher prevalence of fenestrations in the first premolar, posterior teeth, maxillary canines and mandibular molars, similar to the results by Doll [16]. Another interesting finding from our recent survey is the influence of gender on the prevalence of alveolar bone defects, with a higher frequency of fenestrations observed in women than in men. A similar survey by Nalbantoğlu and Yanık [17] found that 80.08% of cases had a buccal bone thickness of ≤1 mm and only 3.66% had a thickness of ≥2 mm. Fenestrations occurred most frequently in canines, with the apical third being the predominant location, while dehiscence was more commonly observed in the coronal third. Furthermore, Nimigean et al. [6] reported high prevalence rates of both fenestration and dehiscence, with 69.56% of skulls showing fenestration and 53.623% showing dehiscence. A higher percentage of fenestration was observed in the maxilla, at 74.679%, while a higher percentage of dehiscence was observed in the mandible, at 71.613%. In a study by Braut et al. [18], they found that 25.70% of the examined teeth had dehiscence and 10.00% had fenestration. In the present study, approximately 5.00% of the cadavers had irregular defects on the estimated teeth. Furthermore, Ghassemian et al. [13] showed that 3.00%–7.50% of maxillary anterior teeth exhibited fenestration and dehiscence was observed in 3.00%–6.10% of these teeth. It is possible that the thinness of the alveolar bone in women contributes to this difference. However, some studies have found no significant difference in the prevalence of bone defects between male and female groups [16]. The present study did not find a significant difference in the frequency of fenestrations and dehiscence between age groups, which is consistent with other studies on the frequency of fenestrations. Similar to the study by Yang et al. [19], no significant differences were observed between age groups. In contrast, some researchers agree [3, 8, 16] that with increasing individual age and increasing prevalence of fenestrations, the incidence of fenestrations in maxillary and canine teeth ranges from 1.20% to 1.20%. Furthermore, Rojo-Sanchis et al. [20] reported that the prevalence of fenestration ranged from 64% to 23.8%; likewise, Kajan et al. [21] found that the fenestration and dehiscence rates were 17.6% and 9.3%, respectively. Cabrejos et al. [22] found that dehiscence occurred in all subjects, with a higher incidence in the mandible (91.6%) and lower canines (100%). Fenestration was observed on the canines in 66.7% of cases, most commonly in the maxilla (28.3%) and on the largest canines (31.7%). Severe cases were more common in dehiscence (65.8%) and fenestration (13.9%), especially in the lower canines (100%) and upper canines (26.7%), respectively, in single bone defects. These results are consistent with the prevalence of dehiscence in our study. An interesting finding of our study is the increased prevalence of fenestration and dehiscence in the smoking group, possibly due to the reduction in cheekbone thickness associated with smoking. However, our results contradict the study by Ghassemian et al. [13], who found no difference in facial bone thickness of the maxillary anterior teeth in smokers. This discrepancy could also be due to the study by Farahmand et al. [23] to be led back, which supports our results and also observed no significant differences in the facial bone thickness of the maxillary anterior teeth between smokers and non-smokers. However, additional studies with larger samples are needed to examine possible significant differences. Additional results from this study demonstrated buccal bone thickness in the anterior maxilla. This is consistent with previous studies by Nowzari et al. [9], Ghassemain et al. [13] and Farhamand et al. [24]. Additional studies are required to explore these bone defects because they are commonly observed in periodontal surgery. It is important to exercise caution and take appropriate measures to prevent and minimise postoperative complications [13, 24, 25]; while, smoking is recognised as a contributing factor to the development of periodontitis, as noted by Bergström, Babcan, and Eliasson [26] and Bahammam [27]. Research has demonstrated that smoking adversely impacts the periodontium, compromising its health. Beyond its detrimental effects on the host's immune response, smoking has also been found to modify the oral microflora, as indicated by Johnson and Hill [28]. The prevailing evidence suggests that smoking plays a crucial role in the inflammation of the marginal periodontium, supported by studies from Johnson and Guthmiller [29], Johnson and Hill [28] and Labriola, Needleman, and Moles [30]. Consequently, it is posited that smoking may similarly influence the health of the apical periodontium [28–33]. Moreover, smoking has been demonstrated to impede healing processes, as highlighted by Pinto et al. [34], and is recognised as a contributing factor to marginal periodontitis, according to Bergström, Babcan, and Eliasson [26]. Nevertheless, our findings suggest a notable difference in the prevalence of bony defects between smokers and non-smokers, which may reflect distinct healing mechanisms at play within the marginal and apical periodontium. The foundation of this research is innovative, necessitating a comprehensive evaluation of the risk factors associated with buccal bone or bony defects, as numerous studies have suggested that smoking does not contribute to an increased prevalence of bony defects [27]. Hence, previous studies on C.B.C.T. have primarily focused on assessing bone morphology, neglecting other aspects such as image quality. Additionally, there is a lack of research exploring the application of C.B.C.T. in evaluating in vivo alveolar bone morphology, which is precisely what this current study aims to address. Instead, most studies have relied on artificially created defects in phantoms or dry skulls, which fail to accurately represent anatomical structures like tooth sockets and alveolar bone margins. The challenge with assessing these artificially created defects in dry skulls is that the detection of defects can be exaggerated due to the subjective interpretation of the operator's limitations [32]. However, Gambarini et al. [33] showed that a total of 68 fenestrations were identified in the maxilla, representing 6.5% of the examined cases. The incisors exhibited the highest frequency of these fenestrations, and the noted prevalence of 11% underscores the importance of conducting C.B.C.T. examinations before any surgical or implant procedures. This precaution is essential to mitigate potential complications arising from the pre-existing fenestrations. The utilisation of C.B.C.T. has proven to be an effective and practical method for the diagnosis of fenestrations. Its application in clinical settings is crucial, as it allows for a comprehensive assessment of the maxillary region, thereby, facilitating informed decision-making in surgical planning and enhancing patient safety during dental interventions [33]. Additionally, the limitations of utilising dried skulls for research include the absence of clinical data and dental history, rendering this method unsuitable for clinical surgical diagnosis [8]. In contrast, both in vivo and *ex vivo* investigations have demonstrated that C.B.C.T. serves as a valuable and more effective clinical instrument for identifying such defects. The advantages of C.B.C.T., including its low radiation exposure and superior image quality compared to traditional computed tomography, position it as the preferred diagnostic tool for assessing fenestration defects [35]. Also, C.B.C.T. technology enables practitioners to analyse the shape and dimensions of the alveolar bone, while circumventing the drawbacks associated with standard radiographic techniques. The images produced through C.B.C.T. are free from distortions and overlaps, enhancing diagnostic accuracy. In the current research, C.B.C.T. scans were employed to assess alveolar bone defects, utilising both axial and transverse sections to provide comprehensive insights into the condition of the bone [36]. The presence of fenestrations and dehiscence in bone defects can disrupt the wound-healing process and increase the likelihood of postoperative complications. Therefore, additional studies are needed to investigate the effects of fenestration and dehiscence on the size and shape of alveolar bone defects as well as their healing process during periodontal surgical therapy. It is essential to explore the frequency and impact of fenestration as well as the factors that contribute to these bone defects and their prevalence in all teeth. The analysis of these risk factors will bring great benefits to our treatment approach and allow us to focus on the occurrence of bone loss, especially with implants and periodontal surgery. Particular attention should be paid to the front teeth area, especially the upper jaw. Before periodontal treatments and implantation procedures, it is important to pay greater attention to the prevalence of bone damage in the maxillary area, which also has aesthetic effects, especially in smoking patients. It is important to note that the results of this study cannot be generalised due to the small sample size and further studies are needed.

## 5. Clinical Relevance

The occurrence of bone defects in the upper and lower jaws has been extensively examined in previous research, yielding valuable outcomes. The interpretation and controversies surrounding the anterior region of the upper jaw and the thickness of the buccal bone plates are particularly noteworthy. These discrepancies become particularly significant in light of the expanding field of periodontal treatments, particularly dental implants, and the emergence of innovative minimally invasive treatment options. Hence, it is essential to consider risk factors like smoking, which can have more significant impacts, to reduce complications post-treatment and enhance long-term prognosis.

## Figures and Tables

**Table 1:** Thicknesses of the buccal bone in the anterior region of the maxilla among males and females who smoker and non-smoker patients.

Gender		Thickness	*N*	Minimum	Maximum	Mean	Standard deviation
Smoker	Female	th4r	102	0.46	1.02	0.7485	0.14293
		th3r	102	0.39	1.04	0.7185	0.11171
		th2r	102	0.34	0.95	0.6854	0.11258
		th1r	102	0.34	0.97	0.6934	0.11875
		th1l	102	0.32	0.97	0.7058	0.11097
		th2l	102	0.29	0.89	0.6831	0.10024
		th3l	102	0.42	1.04	0.7069	0.10617
		th4l	102	0.42	1.04	0.7214	0.12315
	Male	th4r	46	0.60	0.90	0.6886	0.11582
		th3r	46	0.60	0.90	0.6971	0.11800
		th2r	46	0.59	0.67	0.6343	0.03505
		th1r	46	0.54	0.78	0.6671	0.09499
		th1l	46	0.60	0.78	0.6871	0.07588
		th2l	46	0.46	0.67	0.6014	0.06694
		th3l	46	0.60	0.75	0.6586	0.06669
		th4l	46	0.60	0.64	0.6086	0.01574

Non-smoker	Female	th4r	85	0.60	1.04	0.7546	0.10722
		th3r	85	0.60	1.02	0.7329	0.10377
		th2r	85	0.60	1.02	0.7294	0.10457
		th1r	85	0.48	0.98	0.7326	0.09748
		th1l	85	0.45	0.90	0.7194	0.10264
		th2l	85	0.48	1.01	0.7374	0.10779
		th3l	85	0.57	0.97	0.7420	0.10580
		th4l	85	0.60	1.02	0.7403	0.11349
	Male	th4r	67	0.59	0.95	0.6863	0.10730
		th3r	67	0.60	0.95	0.7037	0.10084
		th2r	67	0.60	0.91	0.6873	0.09221
		th1r	67	0.60	0.82	0.6797	0.06830
		th1l	67	0.59	0.89	0.6937	0.07672
		th2l	67	0.57	0.82	0.6660	0.07655
		th3l	67	0.60	0.82	0.6723	0.06388
		th4l	67	0.56	0.85	0.6547	0.07128

**Table 2:** Fenestration rates in maxillary anterior teeth are shown according to gender and smoking habits.

Region	Smoker			Non-smoker		
Right side	Female (%)	Male (%)	*p* value	Female (%)	Male (%)	*p* value
First premolar	57.1	20	0.001	22.5	0	0.003
Canine	42.9	13.3	0.352	15.5	11.4	0.489
Lateral incisor	28.6	36.7	0.932	18.3	17.1	0.034
Central incisor	14.3	6.7	0.933	9.9	14.3	0.717
Left side						
Central incisor	14.3	16.7	0.192	16.9	2.9	0.542
Lateral incisor	28.6	26.7	0.758	19.7	11.4	0.185
Canine	57.1	10	0.018	12.7	5.7	0.019
First premolar	42.9	16.7	0.008	28.2	5.7	0.111

**Table 3:** Frequency of dehiscence in maxillary anterior teeth by gender and smoking habits.

Region	Smoker			Non-smoker		
Right side	Female (%)	Male (%)	*p* value	Female (%)	Male (%)	*p* value
First premolar	14.2	28.6	0.243	16.9	8.36	0.023
Canine	14.3	30	0.760	26.8	20	0.582
Lateral incisor	13.2	14.3	0.550	14.1	11.4	0.705
Central incisor	28.6	10	1	5.6	5.7	0.123
Left side						
First premolar	28.6	13.3	0.262	5.6	2.9	0.033
Canine	14.3	20	0.829	14.1	11.4	0.398
Lateral incisor	14.3	13.3	0.450	19.7	17.1	0.572
Central incisor	14.3	43.3	0.734	15.5	11.4	0.003

**Table 4:** The frequency of fenestration on the right side, as influenced by sex, age, and smoking habits.

Region	Factors	*B*	Standard error	Sig	Exp (*B*)
Right premolar	Age	0.046	0.028	0.107	0/955
	Gender	2.388	0.729	0.001	0.092
	Smoking	2.119	0.704	0.003	8.320

Right canine	Age	0.029	0.028	0.305	1.029
	Gender	0.819	0.560	0.143	0.441
	Smoking	0.818	0.593	0.167	2.267

Right lateral incisor	Age	0.004	0.024	0.881	0.996
	Gender	0.047	0.461	0.919	1.048
	Smoking	0.908	0.427	0.034	2.480

Right central incisor	Age	0.060	0.033	0.068	1.062
	Gender	0.043	0.608	0.944	1.043
	Smoking	0.268	0.745	0.719	0.765

**Table 5:** The frequency of fenestration on the left side, as influenced by sex, age, and smoking habits.

	Factors	*B*	Standard error	Sig	Exp (*B*)
Left premolar	Age	0.023	0.026	0.373	0.978
	Gender	1.243	0.471	0.008	0.289
	Smoking	0.847	0.585	0.148	2.332

Left canine	Age	0.011	0.031	0.717	1.011
	Gender	1.643	0.695	0.018	0.193
	Smoking	1/568	0.670	0.019	4.796

Left lateral incisor	Age	0.023	0.025	0.354	0.977
	Gender	0.437	0.497	0.380	0.646
	Smoking	0.766	0.527	0.146	2.152

Left central incisor	Age	0.060	0.032	0.120	1/046
	Gender	1.226	0.629	0.150	0.462
	Smoking	1.064	0.648	0.100	2.898

**Table 6:** The occurrence of dehiscence on the right side, influenced by sex, age, and smoking behavior.

	Factors	*B*	Standard error	Sig	Exp (*B*)
Left premolar	Age	0.046	0.026	0.084	1.047
	Gender	0.610	0.527	0.247	0.543
	Smoking	1.071	0.470	0.023	2.917

Left canine	Age	0.050	0.024	0.034	1.051
	Gender	0.262	0.442	0.553	0.769
	Smoking	0.373	0.497	0.452	1.453

Left lateral incisor	Age	0.049	0.031	0.116	1.050
	Gender	0.415	0.580	0.475	0.661
	Smoking	0.072	0.675	0.915	1.074

Left central incisor	Age	0.022	0.038	0.559	1.022
	Gender	0.597	0.744	0.422	0.550
	Smoking	1.303	0.754	0.134	3.679

**Table 7:** The frequency of dehiscence on the left side influenced by gender, age, and smoking habits.

	Factors	*B*	Standard error	Sig	Exp (*B*)
Left premolar	Age	0.006	0.025	0.812	1.006
	Gender	0.152	0.488	0.755	1.165
	Smoking	1.306	0.439	0.003	3.693

Left canine	Age	0.044	0.026	0.091	1.045
	Gender	0.285	0.501	0.570	0.752
	Smoking	0.170	0.601	0.777	0.843

Left lateral incisor	Age	0.039	0.028	0.167	1.040
	Gender	0.186	0.540	0.731	0.831
	Smoking	0.610	0.583	0.295	1.841

Left central incisor	Age	0.053	0.039	0.180	0.949
	Gender	0.821	0.784	0.295	0.440
	Smoking	1.712	0.781	0.028	5.538

**Table 8:** The thickness of the buccal bone on the right side is affected by age, gender, and smoking factors.

	Factors	*B*	Standard error	*t*	Sig
Right premolar	Age	0.001	0.001	1.071	0.286
	Gender	0.008	0.024	0.322	0.748
	Smoking	0.070	0.027	2.611	0.010

Right canine	Age	0.001	0.001	1.198	0.233
	Gender	0.016	0.020	0.789	0.431
	Smoking	0.031	0.023	1.362	0.175

Right lateral incisor	Age	0.000	0.001	0.266	0.791
	Gender	0.046	0.019	2.385	0.018
	Smoking	0.046	0.022	2.047	0.043

Right central incisor	Age	0.000	0.001	0.384	0.702
	Gender	0.033	0.019	1.704	0.091
	Smoking	0.044	0.022	1.988	0.049

**Table 9:** The thickness of the buccal bone on the left side, influenced by age, gender, and smoking factors.

	Factors	*B*	Standard error	*t*	Sig
Right premolar	Age	0.001	0.001	0.885	0.378
	Gender	0.026	0.020	1.300	0.196
	Smoking	0.096	0.023	4.162	0.000

Right canine	Age	0.001	0.001	0.755	0.451
	Gender	0.033	0.018	1.799	0.074
	Smoking	0.066	0.021	3.188	0.002

Right lateral incisor	Age	0.001	0.001	0.564	0.573
	Gender	0.058	0.018	3.197	0.002
	Smoking	0.076	0.021	3.701	0.000

Right central incisor	Age	0.001	0.001	0.753	0.453
	Gender	0.014	0.019	0.742	0.459
	Smoking	0.026	0.022	1.218	0.225

## Data Availability

We affirm that we do not oppose the accessibility or the availability of this manuscript following its publication in your journal at any required time, as sharing data and making it available enables researchers to validate the findings of a study, reproduce the analysis and perform additional analyses.
